# Conservative Versus Operative Management of Pediatric Isolated Anterior Shoulder Instability: A Systematic Review and Meta-Analysis

**DOI:** 10.7759/cureus.101704

**Published:** 2026-01-16

**Authors:** Rami Khoshaba, Kate E Hughes, Jordan M Knox, Alexander J Tomesch, Robert Lystrup, Neil L McNinch, Jason L Zaremski, William Denq

**Affiliations:** 1 Sports Medicine, University of Arizona College of Medicine, Tucson, USA; 2 Emergency Medicine, University of Arizona College of Medicine, Tucson, USA; 3 Family and Preventive Medicine, University of Utah Spencer Fox Eccles School of Medicine, Salt Lake City, USA; 4 Primary Care Sports Medicine, Jefferson Einstein Philadelphia Hospital, Philadelphia, USA; 5 Sports Medicine, Tucson Medical Center Healthcare, Tucson, USA; 6 Biostatistics, McNinch Biostats, LLC, Kent, USA; 7 Physical Medicine and Rehabilitation, University of Florida Health, Gainesville, USA; 8 Sports Medicine, University of Florida Health, Gainesville, USA

**Keywords:** anterior shoulder dislocation, arthroscopic bankart repair, pediatric orthopedics, pediatric shoulder instability, recurrent shoulder instability, shoulder subluxation

## Abstract

Anterior shoulder instability, which includes dislocations and subluxations, is a significant concern in pediatric populations (≤19 years). Following an initial dislocation or subluxation, these patients often develop recurrent instability, which can result in long-term functional compromise. While timely and appropriate treatment is crucial, there remains a lack of consensus in the literature regarding the optimal management of pediatric shoulder instability. This systematic review aims to compare the efficacy of conservative and operative treatments for traumatic, isolated anterior shoulder instability in pediatric populations using recurrence of instability and return to play (RTP) at pre-injury levels as outcome measures. This study also provides a contemporary analysis that reflects evolving treatment strategies.

This systematic review targeted studies published between 2013 and 2023 that evaluated pediatric patients (≤19 years), most between 13 and 19 years of age, who received conservative or operative treatment for a first-time or recurrent traumatic isolated anterior shoulder dislocation or subluxation. Statistical analysis was performed to compare rates of recurrence of instability and RTP within each group. Pooled effects (odds ratios) were estimated using fixed-effects models where heterogeneity was absent; otherwise, random-effects models were applied.

A total of 1,459 patients (1,468 shoulders) met the inclusion criteria. Of these, 593 (40.4%) underwent conservative treatment, while 885 (60.3%) received operative interventions. Recurrence rates of instability were higher in the conservative group (236/543, 43.5%) compared to the primary operative group (182/875, 20.8%) and secondary operative group (2/26, 7.7%). RTP rates included 145/193 (75.1%) of conservatively treated patients, 380/480 (79.2%) of primary operative patients, and 8/11 (72.7%) of secondary operative patients returning to pre-injury levels. Among the four studies that directly compared conservative and primary operative treatments, the nonoperative group was more likely to have recurrence compared to the primary operative group (OR = 6.90; 95% CI, 2.28-20.91; p < 0.001). One of these studies was excluded due to methodological differences. A subsequent meta-analysis revealed a significantly higher likelihood of recurrent instability in conservatively treated patients (odds ratio (OR) = 9.55; 95% confidence interval (CI): 5.10-17.88; p < 0.001). In contrast, there was no statistically significant difference in RTP between groups (OR = 3.11; 95% CI: 0.31-30.97; p = 0.33).

The findings support early surgical intervention in pediatric patients to reduce recurrence and improve functional outcomes. Conservative management, while recently shown to be successful for patients with less severe injuries such as subluxations, is still primarily associated with higher recurrence rates compared to operative treatment. Further studies are needed to refine treatment protocols by distinguishing effective strategies for subluxations versus dislocations. Future research should also explore the influence of factors such as sex, skeletal maturity, and activity level in determining optimal management strategies in pediatric patients.

## Introduction and background

Anterior shoulder instability, defined as a dislocation and/or subluxation, is a significant concern among pediatric populations, with dislocations alone occurring at an incidence rate of 60.31 per 100,000 person-years in the United States [1]. These injuries are particularly prevalent among adolescents engaged in contact sports such as American football, rugby, and wrestling [2]. Following the initial instability event, many patients experience recurrent episodes, leading to functional limitations and potential long-term complications such as glenohumeral arthritis [3]. Timely and appropriate treatment is thus essential to reduce recurrent and optimize both short- and long-term outcomes.

Yet, the optimal management of isolated anterior shoulder instability in pediatric populations remains controversial, with no universally accepted standard of care. Historically, surgical intervention was avoided in younger patients due to concerns about damaging the growth plates and the belief that the shoulder could heal spontaneously without operative treatment [4,5]. As such, conservative management, typically involving prompt reduction and immobilization for 1-6 weeks, followed by structural rehabilitation, had long been the mainstay of treatment [6]. Starting in the early 2000s, emerging data have challenged this approach. Studies demonstrated that adolescents, particularly those aged 11-18 years, treated nonoperatively are at significantly higher risk of developing recurrent instability than older individuals or those with closed physes, with reported recurrence rates ranging from 48% to 100% post-treatment [2,4,7].

Advancements in arthroscopic techniques have fueled a growing preference for early surgical intervention in select pediatric cases. Procedures such as arthroscopic or open Bankart repair, capsulolabral reconstruction (with or without remplissage), and the Latarjet procedure have demonstrated improved outcomes and lower recurrence rates compared to conservative care [8]. However, these surgical options are not without limitations. Recurrent instability following arthroscopic Bankart repair has been reported in 5.1%-31% of cases, along with other surgical complications [6,7]. Consequently, early surgical intervention is typically reserved for unidirectional instability following discrete traumatic events, where the underlying structural damage is amenable to anatomical repair. In contrast, multidirectional instability, often associated with ligamentous laxity or repetitive microtrauma, lacks a discrete lesion and is more appropriately managed through rehabilitation and conservative strategies [3,9]. Ultimately, treatment decisions should be individualized, taking into account patient-specific factors such as age, sex, activity level, and mechanism of injury to optimize outcomes while minimizing risk.

To address these ongoing uncertainties, a 2017 systematic review by Zaremski et al. reported significantly higher recurrence rates following nonoperative treatment, particularly in young athletes with first-time dislocations [10]. However, with evolving surgical techniques and broader application in pediatric populations, the most effective treatment strategy continues to be a subject of investigation. This systematic review aims to build upon prior work by evaluating the comparative efficacy of conservative and surgical management for isolated anterior shoulder instability in the broader pediatric population (≤19 years), with a focus on contemporary studies to reflect current clinical practices. Emphasis is placed on identifying strategies that minimize recurrence and improve clinical outcomes in this population.

## Review

Methods

**Figure 1:**
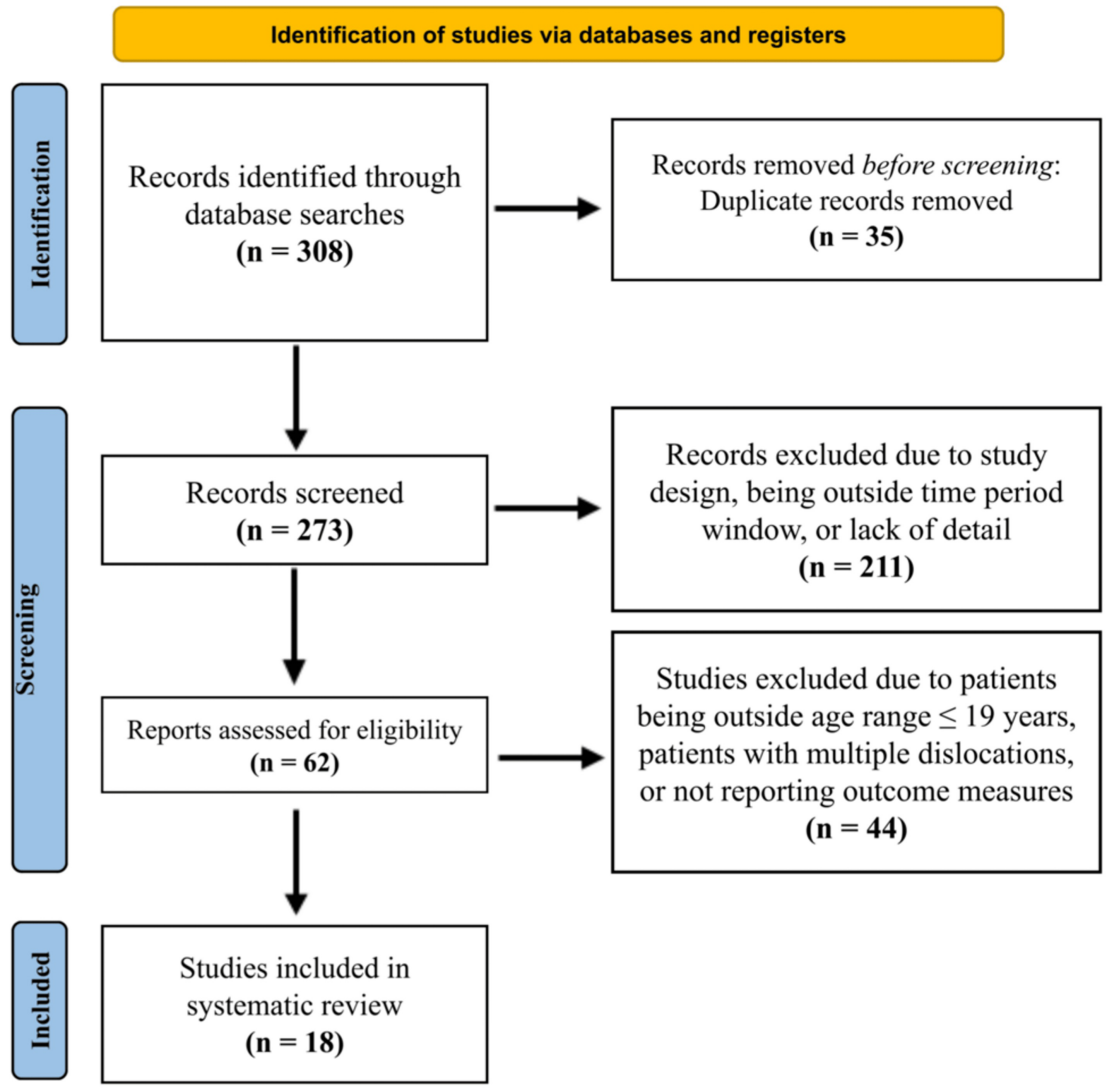
PRISMA flow diagram outlining the study selection process. The figure illustrates the PRISMA 2020 flow of information through the different phases of the systematic review, including identification, screening, eligibility, and inclusion [11]. A total of 308 records were identified, 273 screened, 42 assessed for eligibility, and 18 studies included in the final analysis. PRISMA: Preferred Reporting Items for Systematic Reviews and Meta-Analyses

The systematic review was conducted in accordance with the Preferred Reporting Items for Systematic Reviews and Meta-Analyses (PRISMA) 2020 (Figure 1) [11]. Six databases were extensively searched, including Ovid EMBASE, Web of Science, PubMed, Google Scholar, Scopus, and Cochrane, between September 2023 and December 2023, with a 10-year timeframe restriction. This timeframe (2013-2023) was selected to provide a contemporary synthesis reflecting modern arthroscopic techniques, evolving surgical indications, and current rehabilitation protocols. The search algorithm utilized Boolean operators combining population terms using OR (e.g., “pediatric,” “adolescent,” and “teenager”) to maximize sensitivity, along with condition-specific terms related to anterior shoulder instability, including “anterior shoulder instability,” “anterior shoulder subluxation,” and “anterior shoulder dislocation.” For the purposes of this review, the term “pediatric” refers to patients aged 19 years and younger, consistent with the age group reported across included studies to ensure consistency when evaluating outcomes. Following the search, relevant studies were compiled and uploaded to Mendeley reference management software. Abstract screening and eligibility assessment were performed by the author (RK).

Inclusion and Exclusion Criteria

The inclusion criteria encompassed randomized controlled trials, prospective and retrospective cohort studies, case-controlled studies, cross-sectional studies, and case series with at least three patients. Eligible studies were required to be published in English within the past 10 years in a peer-reviewed journal. Studies were excluded if they did not meet the inclusion criteria or failed to align with the Population, Intervention, Comparison, and Outcome (PICO) framework. Specifically, the population included pediatric patients (≤19 years) treated for isolated anterior shoulder instability; the intervention was operative management; the comparison group received conservative management; and the primary outcomes following initial treatment included recurrence of injury following initial management, as well as return to play (RTP) of the sport participated in at pre-injury levels. Operative interventions were further subdivided into primary and secondary interventions: primary operative treatment was defined as surgical intervention on a native (previously unoperated) shoulder, whereas secondary operative treatment referred to the surgical intervention following failure of a prior surgical procedure.

Risk of Bias Assessment

The primary instrument used to assess methodological quality and risk of bias in this review was a modified version of the Coleman Methodology Score (CMS), adapted from the framework established by Zaremski et al. [10]. Each study was evaluated across 11 categories, receiving points based on predefined criteria for a maximum possible score of 100. A high CMS score indicates that the study largely avoids chance, various biases, and confounding factors. Interrater reliability was found to be moderate to excellent, with an intraclass correlation coefficient (ICC) of 0.83 (95% confidence interval (CI): 0.65-0.93), based on the interpretive criteria outlined by Koo et al. [12]. Statistical analysis further supported rejection of the null hypothesis that the ICC equals zero (F(18,54) = 4.61, p < 0.001).

The assessed categories included study design, study size, age of participants, description of diagnosis, description of management, follow-up duration, description of rehabilitation (for conservative treatment), description of postoperative rehabilitation (for operative management), outcome reporting, selection criteria, and procedure assessment recruitment. A complete list of the scoring criteria is presented in Table 1, while individual study scores are summarized in Table 2. Four authors (KH, JK, AT, and ML) independently reviewed and scored each study, with discrepancies resolved by consensus.

**Table 1:** Modified CMS criteria and scoring breakdown Detailed breakdown of the modified CMS scoring system used to evaluate study quality in this review [10]. Part A assesses study design, patient demographics, and treatment description (maximum of 60 points). Part B evaluates outcomes, selection criteria, and recruitment methods (maximum of 40 points). Total scores range from 0 to 100, with higher scores indicating greater methodological rigor. CMS: Coleman Methodology Score

Part A: Only one score to be given for each of the seven sections	Score
1. Study design	
Randomized controlled trial	15
Prospective cohort	7
Retrospective cohort	0
2. Study size: number of shoulders	
N > 60	10
N = 41-60	7
N = 20-40	4
N < 20 or unknown	0
3. Age	
<18 (average age and distribution)	10
<18 (average age only)	5
>18 years	0
4. Description of diagnosis	
Dislocation as diagnosis, percentage specified for each type	5
Dislocation as diagnosis, without percentage breakdown	0
5. Description of management	
Adequate (single approach, detailed description)	10
>1 approach, outcomes reported separately	7
Inadequate or unclear	0
6. Follow-up period	
>24 months	10
12-24 months	5
<12 months	0
7. Description of rehabilitation (if nonoperative alone)	
Well-described with patient compliance	5
Not reported or no compliance	0
8. Description of postoperative rehabilitation	
Well-described with patient compliance	5
Not reported or no compliance	0
Part B: Scores may be given for each option in each of the three sections if applicable	
1. Outcomes	
Outcome measures clearly defined	3
Timing of outcome assessment clearly stated	3
Use of outcome criteria has reported reliability	4
2. Selection criteria report and unbiased	
Selection criteria reported and unbiased	5
Recruitment rate reported >80%	5
3. Procedure assessing recruitment	
Subjects recruited	5
Investigator independent of practitioner	4
Written/electronic assessment	3
Assessment with minimal investigator assistance	3

**Table 2:** Risk of bias assessment based on the modified CMS Summary of CMS values assigned to the 19 included studies [13-30]. Individual reviewer scores (A-D), along with average and standard deviation, are shown. Higher CMS scores indicate greater methodological rigor and lower risk of bias. CMS: Coleman Methodology Score

Study author	Year	Total CMS score (A)	Total CMS score (B)	Total CMS score (C)	Total CMS score (D)	Total CMS score (average)	Total CMS score (SD)
Khan et al. [13]	2014	63	64	58	50	58.75	6.4
Gigis et al. [14]	2014	80	72	80	78	77.5	3.79
Roberts et al. [15]	2015	62	57	54	54	56.75	3.77
Shymon et al. [16]	2015	57	54	54	64	57.25	4.72
Saper et al. [17]	2017	67	64	58	52	60.25	6.65
Hatch et al. [18]	2018	59	44	57	65	56.25	8.85
Torrance et al. [19]	2018	67	69	66	52	63.5	7.77
Cordasco et al. [20]	2020	73	63	73	73	70.5	5
Shanley et al. [21]	2019	56	41	61	59	54.25	9.07
Kramer et al. [22]	2019	51	55	51	54	52.75	2.06
Tokish et al. [23]	2020	45	47	59	40	47.75	8.06
Cheng et al. [24]	2021	39	56	44	61	50	10.23
Hickey et al. [25]	2022	61	62	67	47	59.25	8.58
Novakofski et al. [26]	2022	41	51	34	38	41	7.26
Waltenspül et al. [27]	2022	53	47	63	52	53.75	6.7
Kelley et al. [28]	2022	60	48	62	51	55.25	6.8
Egger et al. [29]	2023	64	59	72	65	65	5.35
Harada et al. [30]	2023	62	55	58	45	55	7.26

Given the predominance of retrospective study designs and moderate CMS scores, findings from this review, particularly those favoring operative management, should be interpreted as associative rather than causal. Quality scores were therefore used to contextualize the strength of the evidence rather than to weight or exclude studies, and conclusions were framed conservatively to reflect the underlying risk of selection and information bias.

Meta-Analysis

Meta-analysis was conducted using STATA (StataCorp LLC, College Station, TX). Random-effects models were designated a priori as the primary analytical approach given anticipated clinical and methodological heterogeneity across studies. Fixed-effects models were used only as sensitivity analyses when heterogeneity was minimal. Heterogeneity was assessed using Higgins’ I² statistic. Evaluation of publication bias included visual examination of funnel plots, both regular and contour enhanced, as well as conduct of the Egger’s regression test to detect small-study effects. The threshold for statistical significance is established a priori as alpha = 0.05.

Results

The initial search strategy yielded 308 articles, of which 246 manuscripts were excluded due to duplication or not matching the PICO criteria. The remaining 62 manuscripts were individually reviewed by authors RK and WD. for eligibility. Following full-text screening, 18 studies were included in the final analysis [13-30]. These consisted of nine retrospective reviews, six case series, and three prospective studies. Risk of bias was assessed using the modified Coleman Methodology Score, with average study scores ranging from 41 to 77.5 (mean: 57.5 ± 8.1). Relevant data were extracted from the included full text, tables, and figures and compiled into a spreadsheet for analysis, as shown in Table 3.

**Table 3:** Summary of included studies Summary of study characteristics, including total number of shoulders, patient sex distribution, treatment modality (conservative versus operative), mean age, and CMS for methodological quality assessment. Data extracted from included studies [13-30]. CMS: Coleman Methodology Score

Author	Total shoulders (number)	Male (number)	Females (number)	Conservative (number)	Operative (number)	Mean age (years)	Total CMS score
Khan et al. [13]	53	35	14	25	28	15.9	58.8
Gigis et al. [14]	65	41	24	27	38	16.8	77.5
Roberts et al. [15]	133	115	18	133	60	16.3	57.3
Shymon et al. [16]	99	-	-	99	-	16.9	56.8
Saper et al. [17]	39	38	1	39	-	16.9	60.3
Hatch et al. [18]	20	19	2	20	-	16	56.3
Torrance et al. [19]	67	66	1	16	-	16.3	63.5
Cordasco et al. [20]	67	48	19	-	67	17.4	54.3
Shanley et al. [21]	129	108	21	97	32	15.8	52.8
Kramer et al. [22]	36	-	-	-	33	16	70.5
Tokish et al. [23]	57	47	10	57	-	15.8	47.8
Cheng et al. [24]	171	141	30	171	-	15.9	50
Hickey et al. [25]	66	44	22	77	-	16.4	59.3
Novakofski et al. [26]	261	183	71	261	-	-	41
Waltenspül et al. [27]	34	34	0	34	-	-	53.8
Kelley et al. [28]	62	52	10	62	-	18.7	55.3
Egger et al. [29]	59	46	13	59	-	-	55
Harada et al. [30]	50	35	15	50	-	16.8	65
Total number or mean (SD)	1468	1052	272	593	885	16.5 (0.7)	57.5 (8.1)

Among the 18 included studies, a total of 1,468 shoulders from 1,459 patients were analyzed. The majority of patients were male, comprising approximately 1,052/1,334 (78.9%), while female patients accounted for 272/1,334 (20.3%). Of note, two studies, Shymon et al. [16] and Kramer et al. [22], did not specify patient sex. Of the total cohort, 593/1,468 (40.4%) underwent conservative treatment, while 885/1,468 (60.2%) received operative treatment for anterior shoulder instability. Operative interventions included 657 (74.2%) arthroscopic Bankart repairs, 82 (9.3%) open Bankart repairs, 65 (7.3%) open Latarjet procedures, 16 (1.8%) arthroscopic anterior labral repairs, 6 (0.7%) remplissage procedures, and 59 (6.7%) unspecified procedures.

Recurrence of instability was reported in 236/543 (43.5%) patients treated conservatively, 182/875 (20.8%) patients treated with a primary operative intervention, and 2/26 (7.7%) patients who underwent a secondary operative procedure (Table 4). Return to play (RTP), defined as the patient’s ability to return to pre-injury level sport, was achieved in 145/193 (75.1%) conservatively treated patients, 380/480 (79.2%) primary operative patients, and 8/11 (72.7%) secondary operative patients, as shown in Table 5. However, the interpretation of RTP outcomes is limited by inconsistent reporting and delayed assessment in retrospective studies (i.e., two-year follow-up in Saper et al. [17]), which may overestimate operative success. Additionally, RTP timing, such as return within the same season versus long-term, was often unspecified, further limiting comparability. Due to limited data, subgroup analyses by age or sex were not performed.

**Table 4:** Recurrence rates by treatment type Recurrence of instability stratified by treatment type: primary conservative, primary operative, and secondary operative interventions. Frequencies reflect the proportion of recurrent instability events relative to the total number of patients in each treatment group. Data extracted from included studies [13-30].

Author (year)	Recurrence rate					
	Conservative		Primary operative		Secondary operative	
	Events	Total	Events	Total	Events	Total
Khan et al. [13]	14	25	2	28	-	-
Gigis et al. [14]	19	27	5	38	-	-
Roberts et al. [15]	102	133	19	60	0	7
Shymon et al. [16]	-	-	14	46	-	-
Saper et al. [17]	-	-	4	39	-	-
Hatch et al. [18]	-	-	0	20	-	-
Torrance et al. [19]	-	-	4	67	-	-
Cordasco et al. [20]	-	-	4	67	-	-
Shanley et al. [21]	6	97	2	32	-	-
Kramer et al. [22]	-	-	9	36	-	-
Tokish et al. [23]	-	-	-	-	-	-
Cheng et al. [24]	-	-	35	171	-	-
Hickey et al. [25]	-	-	22	66	2	19
Novakofski et al. [26]	95	254	-	-	-	-
Waltenspül et al. [27]	-	-	8	34	-	-
Kelley et al. [28]	-	-	4	62	-	-
Egger et al. [29]	-	-	18	59	-	-
Harada et al. [30]	-	-	2	50	-	-
Totals	236	543	182	875	2	26
Frequency	-	43.46%	-	20.8%	-	7.69%

**Table 5:** RTP rates by treatment type RTP outcomes across conservative, primary operative, and secondary operative interventions. Full RTP is defined as the ability to return to pre-injury sport participation. Frequencies represent the percentage of patients in each group who achieved full RTP relative to their respective total. Data extracted from included studies [13-30]. RTP: return to play

Author (Year)	Return to play					
	Conservative		Primary operative		Secondary operative	
	Full RTP	Total	Full RTP	Total	Full RTP	Total
Khan et al. [13]	3	20	21	26	-	-
Gigis et al. [14]	15	19	25	27	-	-
Roberts et al. [15]	-	-	-	-	-	-
Shymon et al. [16]	-	-	-	-	-	-
Saper et al. [17]	-	-	25	32	-	-
Hatch et al. [18]	-	-	20	20	-	-
Torrance et al. [19]	-	-	-	-	-	-
Cordasco et al. [20]	-	-	50	67	-	-
Shanley et al. [21]	82	97	23	32	8	11
Kramer et al. [22]	-	-	24	29	-	-
Tokish et al. [23]	45	57	-	-	-	-
Cheng et al. [24]	-	-	-	-	-	-
Hickey et al. [25]	-	-	27	42	-	-
Novakofski et al. [26]	-	-	-	-	-	-
Waltenspül et al. [27]	-	-	27	34	-	-
Kelley et al. [28]	-	-	62	62	-	-
Egger et al. [29]	-	-	38	59	-	-
Harada et al. [30]	-	-	38	50	-	-
Totals	145	193	380	480	8	11
Frequency	-	75.13%	-	79.17%	-	72.72%

**Figure 2:**
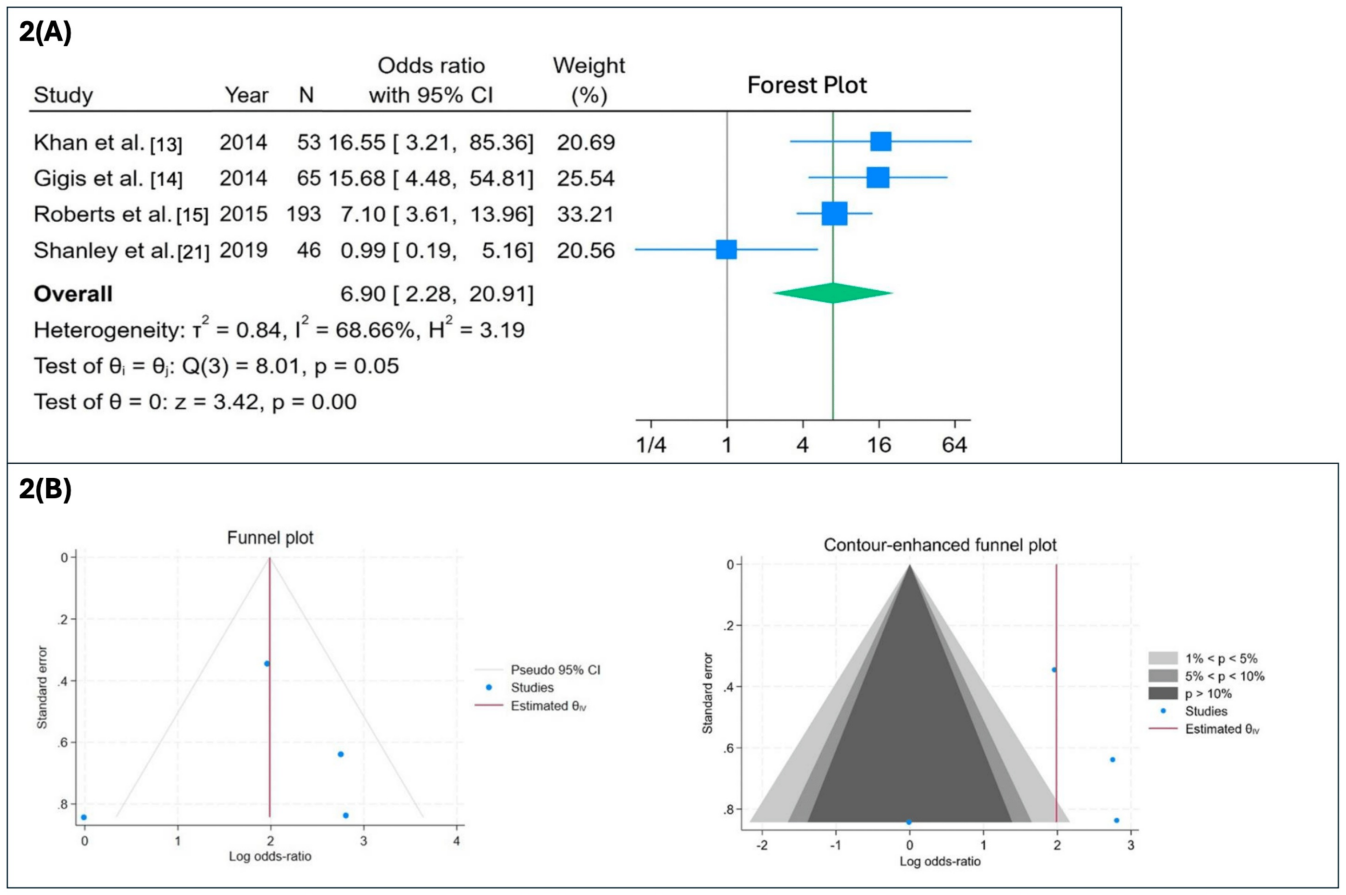
Meta-analysis of recurrence rates following conservative versus operative management (random-effects model) (A) Forest plot of ORs with 95% CIs from four studies (Khan et al. [13], Gigis et al. [14], Roberts et al. [15], and Shanley et al. [21]) comparing recurrence rates following conservative versus primary operative treatment. Conservative management was associated with a significantly higher recurrence rate (OR = 6.90; 95% CI: 2.28-20.91; p < 0.001). Moderate heterogeneity was observed (I² = 68.7%). (B) Standard funnel and contour-enhanced funnel plots assessing publication bias. Funnel-plot asymmetry suggested possible publication bias; however, Egger’s test did not reach statistical significance (p = 0.734). OR: odds ratios, CI: confidence interval

Three meta-analyses were performed to compare the incidence of recurrence and return to play in conservative versus primary operative treatment outcomes in the patient populations. Among the four qualifying studies (Khan et al. [13], Gigis et al. [14], Roberts et al. [15], and Shanley et al. [21]) that directly compared conservative and operative interventions, patients in the primary nonoperative group were significantly more likely to experience recurrence compared to those in the primary operative group (OR = 6.90; 95% CI: 2.28-20.91; p < 0.001). However, visual inspection of funnel plots and contour-enhanced funnel plots revealed asymmetry and a lack of small studies in regions of nonsignificance, suggesting potential publication bias, although Egger’s regression test for small-study effects was not statistically significant (Figure 2).

**Figure 3:**
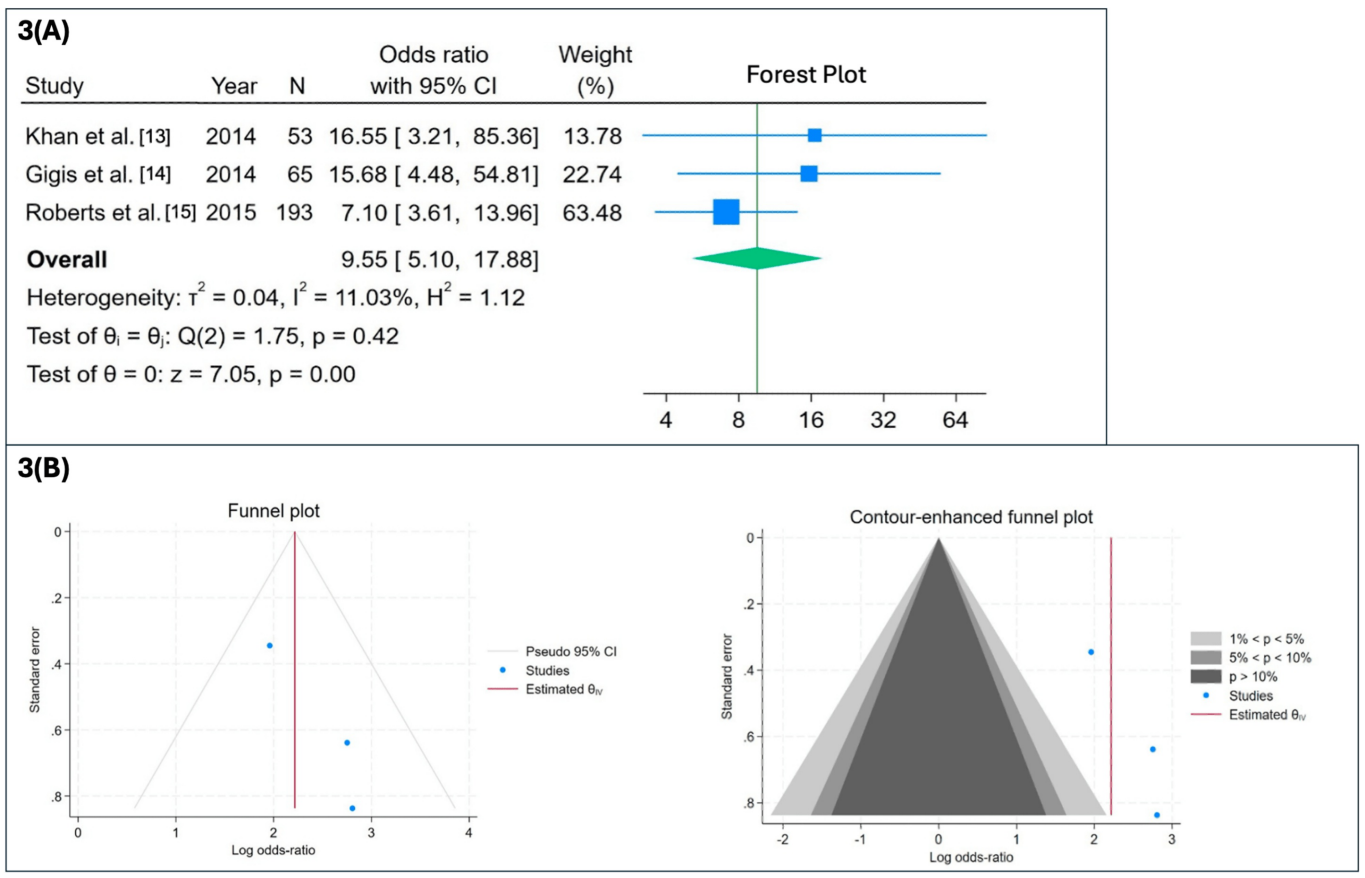
Meta-analysis of recurrence rates following conservative versus operative management (fixed-effects model; Shanley et al. was excluded) (A) Forest plot summarizing ORs with 95% CIs from three studies (Khan et al. [13], Gigis et al. [14], and Roberts et al. [15]) comparing recurrence rates following conservative versus primary operative treatment. Exclusion of Shanley et al. [21] demonstrated a stronger association favoring surgical treatment (OR = 9.55; 95% CI: 5.10-17.88; p < 0.001) with low heterogeneity (I² = 11.03%). (B) Standard funnel and contour-enhanced funnel plots assessing publication bias. Visual asymmetry suggests possible publication bias; however, Egger’s regression test did not reach statistical significance (p = 0.202). OR: odds ratios, CI: confidence interval

A predefined sensitivity analysis excluding Shanley et al. [21] was performed. In this fixed-effects sensitivity analysis, exclusion of Shanley et al. [21] reduced heterogeneity and yielded a stronger association favoring operative management (OR = 9.55; 95% CI: 5.10-17.88; p < 0.001) (Figure 3). This sensitivity analysis was not used as the basis for conclusions, and all primary inferences were drawn from random-effects models including all eligible studies.

**Figure 4:**
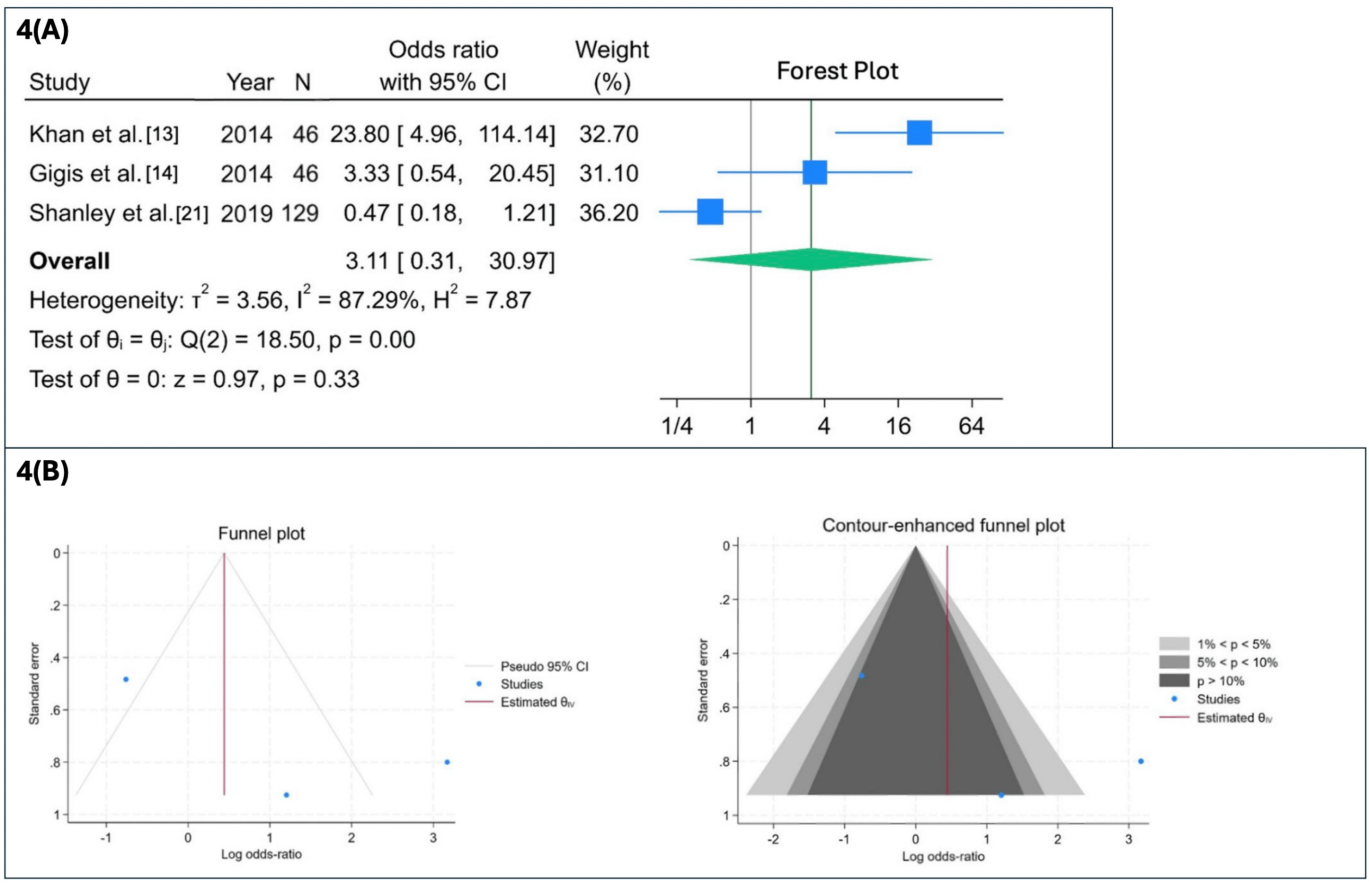
Return to play after conservative versus operative treatment (random-effects model) (A) Forest plot summarizing ORs with 95% CIs from three studies (Khan et al. [13], Gigis et al. [14], and Shanley et al. [21]) comparing return-to-play rates following conservative versus operative treatment. No statistically significant difference was observed between treatment groups (OR = 3.11; 95% CI: 0.31-30.97; p = 0.33); however, substantial heterogeneity was present (I² = 87.3%), and the limited number of included studies and variability in return-to-play definitions preclude definitive conclusions. (B) Standard funnel and contour-enhanced funnel plots assessing potential publication bias. Interpretation is limited by the small number of included studies. OR: odds ratios, CI: confidence interval

The second random-effects meta-analysis compared RTP rates across three studies (Khan et al. [13], Gigis et al. [14], and Shanley et al. [21]). Substantial heterogeneity was present (I² = 87.3%), reflecting variability in RTP definitions, follow-up duration, sport-specific demands, and study design (Figure 4). Given the limited number of included studies, these findings should be interpreted as exploratory and descriptive rather than confirmatory due to anticipated variability in outcome definitions and assessment timelines.

Discussion

The findings of this systematic review are consistent with those reported by Zaremski et al., demonstrating that primary nonoperative management of anterior shoulder instability in pediatric patients is associated with higher recurrence rates compared with operative intervention [10]. This association remained consistent across analytical approaches and was supported by random-effects meta-analysis. Sensitivity analyses were performed to evaluate the impact of methodological heterogeneity on pooled estimates.

Rationale for Exclusion of Shanley et al.

Shanley et al. reported comparable recurrence rates between young athletes treated operatively and those managed nonoperatively [21]. A likely explanation for this finding lies in the study’s definition of recurrence, whereby athletes who experienced recurrent instability but continued sport participation without time loss were classified as treatment successes. In contrast, most studies in the broader literature defined recurrence as any subsequent instability episode regardless of its impact on playing time. This difference in classification may have led to an underestimation of the actual recurrence rate, particularly among nonoperatively managed patients with subluxations who may continue to compete despite ongoing instability symptoms.

Accordingly, exclusion of Shanley et al. from the fixed-effects sensitivity analysis was driven by methodological considerations rather than outcome direction. Inclusion of this study in a fixed-effects model introduced clinical heterogeneity that violated model assumptions, whereas retention in the random-effects analysis preserved methodological transparency and allowed for the comprehensive synthesis of the available evidence.

Outcomes Following Conservative Treatment

Historically, conservative management has been associated with favorable outcomes and lower recurrence rates in skeletally immature patients (<14 years), largely due to the inherent stability provided by compliant capsuloligamentous structures and rapid tissue healing potential in this age group [4-6,31]. However, recurrence risk rises sharply during mid- to late adolescence as physeal closure and participation in contact or overhead sports increase mechanical stress on the glenohumeral joint [9,20]. To better identify which adolescents might succeed with nonoperative care, Tokish et al. introduced the Nonoperative Instability Severity Index Score (NISIS) as a tool to help predict nonoperative treatment success and facilitate more informed decision-making in the management of adolescent shoulder instability [23]. They reported that 97% of “low-risk” patients (age < 15, no bone loss, and subluxation injury) were able to return to sport following conservative treatment, compared to only 59% of their “high-risk” counterparts (age > 15, bone loss, and dislocation injury) [23]. While NISIS offers a valuable tool for risk stratification, it should be applied in conjunction with clinical judgment rather than as a sole determinant of care.

Despite these efforts, several studies have questioned the long-term effectiveness of conservative management for anterior shoulder instability. Roberts et al. found that neither sling immobilization nor physical rehabilitation effectively reduced recurrence rates in adolescent patients with primary anterior shoulder dislocation, with a recurrence rate of 72.7% at two-year follow-up (p < 0.001) [15]. Similarly, Novakofski et al. observed high rates of recurrent instability (37.5%), persistent shoulder pain (58%), and symptomatic osteoarthritis (12.2%) among conservatively treated adolescents [26]. Although widely used, the optimal duration of immobilization and rehabilitation remains undefined, highlighting the need for further evidence-based treatment protocols. Collectively, these findings reinforce that management should be individualized and patient-centered, with treatment decisions tailored to skeletal maturity, activity level, and recurrence risk rather than guided by a uniform approach.

Outcomes Following Operative Management

While nonoperative measures were once preferred for managing primary dislocations in pediatric patients, recent studies suggest that surgical interventions may lead to lower recurrence rates following stabilization. Gigis et al. found a significant difference in recurrence rates between nonoperatively treated patients (70%) and operatively treated ones (13%), highlighting the benefits of surgical stabilization procedures [14]. Similarly, a recent cost-effective analysis by Oeding et al. demonstrated that arthroscopic Bankart repair not only significantly reduces recurrence but is also highly cost-effective over a 10-year period for a broader population of young individuals aged 12-26 [32]. Despite representing different age groups, these findings reinforce the trends observed in our pediatric cohort and support the growing preference for early operative management in appropriate cases.

Although arthroscopic techniques account for 87%-91% of surgical shoulder stabilization procedures in the United States, the most appropriate approach for pediatric patients varies depending on patient-specific factors and surgeon preference [33]. Saper et al. demonstrated the effectiveness of arthroscopic Bankart repair for traumatic instability; however, they also reported higher recurrence rates among younger patients with an overall recurrence rate of 27.9% that increased to 40.7% in patients aged 13-16 years [17]. Similarly, Torrance et al. [19] and Cordasco et al. [20] observed a 51% recurrence rate in adolescents undergoing arthroscopic labral repairs following significant contact-related shoulder injuries, reinforcing concerns about higher failure rates in this subgroup. In contrast, Khan et al. [13] and Waltenspül et al. [27] advocated for the use of the Latarjet procedure in cases involving recurrent dislocations, particularly in the setting of high Instability Severity Index Scores (ISIS) or significant glenoid bone loss. Kramer et al. [22] noted higher recurrence rates among patients treated with Bankart repair alone compared to those who underwent a Bankart and remplissage procedure, suggesting that augmentation may improve outcomes in select high-risk patients [34]. 

Regardless of the surgical technique, postoperative rehabilitation plays a critical role in minimizing the risk of recurrent instability. Khan et al. [13] emphasized the importance of immobilization followed by a structured rehabilitation program, while Saper et al. [17] outlined a five-stage recovery protocol. Kelley et al. proposed a program that incorporates both functional testing and psychological readiness to ensure that young athletes are fully prepared to return to sports while minimizing recurrence [28]. The protocol by Hickey et al. permitted return to contact sports after 12 weeks of intensive rehabilitation, emphasizing a gradual, staged recovery process [25].

Risk Factors for Recurrent Instability

Adolescents with anterior shoulder instability are at increased risk for recurrence, as only 7% remained stable 10 years post-initial dislocation, with a higher postoperative dislocation rate in adolescents compared to adults [15]. This heightened risk is further influenced by several clinical features that have been associated with failure following arthroscopic repair, including younger age, a greater number of preoperative dislocations, delayed surgical intervention, off-track lesions, large Hill-Sachs lesions, glenoid bone loss, bilateral instability, prior closed reductions, and generalized ligamentous laxity [22].

In contrast to the general pediatric population (≤19 years), young athletes typically engage in high-intensity, repetitive overhead, and/or contact activities that place significant stress on the shoulder joint. These elevated functional demands contribute to a heightened risk of recurrent instability and often shift the treatment paradigm in favor of operative interventions over conservative approaches [20]. Given the potential for structural damage associated with recurrent dislocations, primary surgical intervention may optimize an athlete’s chances of returning to sport at full capacity while minimizing the risk of further injury to both bony and soft tissue structures [25]. Furthermore, Shymon et al. reported a 30.4% recurrence rate in athletic pediatric populations and attributed the high failure rate to premature return to physical activity [16]. Supporting this, Zaremski et al. concluded that young athletes (<14 years) treated nonoperatively were significantly more likely to have recurrence compared to the primary operative group (OR = 13.41; 99% CI: 3.60-49.93; p < 0.001) [10]. Consistent with these findings, our meta-analysis demonstrated a similar trend among young patients in the general population (OR = 9.55; 95% CI: 5.10-17.88; p < 0.001), supporting early surgical intervention in pediatric patients with high-risk factors such as early age at initial dislocation and presence of structural abnormalities that predispose to recurrent instability.

Return-to-Play Outcomes and Considerations

Return to play (RTP) is a clinically meaningful outcome in young athletes with anterior shoulder instability; however, its interpretation is closely linked to injury severity and functional status at presentation. The Shanley study highlights this relationship, demonstrating that athletes who sustained a dislocation were significantly less likely to return to play than those experiencing a subluxation (26% versus 89%, p = 0.013) [21]. This difference reflects the greater structural damage and functional impairment associated with dislocations, which may increase the risk of recurrent instability and the need for surgical intervention. In contrast, patients with less severe instability, such as subluxations, may be more likely to return to sport and benefit from structured nonoperative rehabilitation. Accordingly, RTP outcomes should be interpreted in the context of injury severity and clinical recovery, underscoring the need for individualized treatment planning and cautious interpretation of pooled RTP analyses.

In light of these considerations, the random-effects meta-analysis evaluating return-to-play outcomes did not demonstrate a statistically significant difference between operative and nonoperative management. Substantial heterogeneity was observed (I² = 87.3%), reflecting variability in RTP definitions, follow-up duration, sport-specific demands, and study design across included studies. Additionally, athletes, particularly those treated nonoperatively, may return to sport despite persistent instability, apprehension, or activity modification, potentially inflating reported RTP rates and obscuring meaningful functional deficits. Given the limited number of included studies and high heterogeneity, RTP outcomes should be interpreted as descriptive and hypothesis-generating rather than definitive indicators of treatment efficacy.

Limitations

Several limitations should be acknowledged. Many included studies were retrospective in design and demonstrated moderate methodological quality as assessed by the modified Coleman Methodology Score, introducing potential selection and information bias. Additionally, the generalizability of these findings is limited by the relatively small number of eligible published studies within the 10-year timeframe (2013-2023). Comparative analysis of recurrence or return-to-play outcomes across excluded studies was not performed, as insufficient data precluded meaningful assessment of heterogeneity and bias, rendering such comparisons unreliable.

Notably, this analysis included both dislocations and subluxations, which may introduce bias, as subluxations may be more amenable to nonoperative management than dislocations. Furthermore, heterogeneity in outcome definitions, rehabilitation protocols, and follow-up duration limited the ability to perform subgroup analyses based on age, sex, skeletal maturity, sport type, or injury characteristics (e.g., first-time versus recurrent events).

Future studies with larger, prospectively collected cohorts are needed to better evaluate these subgroups and identify patients at increased risk for recurrence, thereby informing more individualized treatment strategies. Additionally, future research should aim to directly compare conservative and operative management in cohorts that include both dislocations and subluxations. To improve consistency and comparability across studies, standardized and clinically meaningful definitions of recurrence and return to play should be adopted.

## Conclusions

Operative management is associated with lower recurrence rates in pediatric patients with isolated anterior shoulder instability, whereas nonoperative treatment may be appropriate in carefully selected cases such as isolated subluxation events. Although causal conclusions cannot be drawn, the available evidence consistently demonstrates higher recurrence rates following conservative management compared with operative intervention. Management decisions should therefore be individualized, incorporating patient-specific risk factors, skeletal maturity, activity demands, and patient preferences to optimize functional outcomes.
